# Detection of Salivary Insulin Following Low versus High Carbohydrate Meals in Humans

**DOI:** 10.3390/nu9111204

**Published:** 2017-11-02

**Authors:** Étienne Myette-Côté, Katie Baba, Raj Brar, Jonathan Peter Little

**Affiliations:** 1 School of Health and Exercise Sciences, University of British Columbia Okanagan, 3333 University Way, Kelowna, BC V1V 1V7, Canada; emyco@alumni.ubc.ca; 2 Southern Medical Program, University of British Columbia Okanagan, 3333 University Way, Kelowna, BC V1V 1V7, Canada; katiembaba@hotmail.com (K.B.); rbrar1992@gmail.com (R.B.)

**Keywords:** saliva, insulin, postprandial, low-carbohydrate, high-fat, hyperinsulinemia

## Abstract

Developing non-invasive alternatives to monitor insulin levels in humans holds potential practical value for identifying individuals with, or at risk of developing, insulin resistance. The aims of this study were: (1) to determine if saliva insulin can be used to delineate between low and high postprandial insulin levels following the ingestion of mixed breakfast meals; and (2) to determine if expected differences in postprandial hyperinsulinemia between young lean and young overweight/obese participants could be detected in saliva. Sixteen individuals (*n* = 8 classified as normal weight (NW); BMI 20.0–24.9 kg/m^2^, and *n* = 8 classified as overweight/obese (OO); BMI ≥ 28.0 kg/m^2^) completed two isocaloric mixed-meal tolerance tests following an overnight fast, consisting of a low-carbohydrate (LC) breakfast or a high-carbohydrate (HC) breakfast. Blood and saliva samples were collected at regular intervals for two hours postprandially. In both groups, plasma and saliva insulin total area under the curve (AUC) and incremental AUC (iAUC) were significantly higher after the HC as compared to the LC meal (all *p* ≤ 0.005). Insulin AUC and iAUC in both plasma and saliva were higher in OO than in NW after the HC meal (all *p* ≤ 0.02) but only plasma and saliva total AUC were higher in OO after the LC meal (both *p* ≤ 0.01). Plasma insulin AUC was significantly correlated with salivary insulin AUC in LC (*r* = 0.821; *p* < 0.001) and HC (*r* = 0.882; *p* < 0.001). These findings indicate that saliva could potentially be used to delineate between low and high insulin levels following mixed breakfast meals.

## 1. Introduction

Compelling data show that elevated insulin levels are associated with the development of pathological conditions such as obesity and type 2 diabetes [1]. For the past few decades, hyperinsulinemia has been mainly considered as a consequence of obesity but this concept has recently been revisited [2]. A revised model of obesity and type 2 diabetes suggests a more central and causal role of hyperinsulinemia, which is thought to precede and drive metabolic abnormalities [3,4]. Insulin hypersecretion and insulin resistance are detectable up to several years prior to abnormalities in glucose tolerance [5,6,7,8,9]. Over time, persistent elevated insulin secretion can no longer be maintained by pancreatic beta-cells, leading to chronic hyperglycemia and the associated diagnoses of prediabetes or type 2 diabetes [10], which significantly increases the risk for cardiovascular disease and mortality [11]. Thus, elevations in basal and stimulated insulin levels may constitute an important early marker of metabolic dysfunction that could be monitored in both apparently healthy and at-risk individuals.

The theoretical and practical importance of postprandial insulin levels have previously been emphasized by proposing the use of a food insulin index that ranks foods based on their ability to elevate postprandial insulin [12,13]. Although potentially useful, a general food insulin index based on the postprandial blood insulin responses to isolated foods in healthy volunteers [12] would not take into account the inter-individual variability in insulin secretion and/or responses to mixed meals nor would it be able to account for expected differences in postprandial insulin levels between individuals with different levels of insulin resistance. From a clinical and monitoring perspective, measuring postprandial insulin levels would be highly valuable but presents several challenges, including the requirement for repeated blood sampling. Developing non-invasive, user-friendly alternatives to monitor insulin levels in humans therefore holds potential value.

As a body fluid, saliva contains several of the same components as blood, but can be sampled non-invasively. In clinical research settings, saliva is already being used as a diagnostic and/or monitoring tool to reflect the content of circulating hormones, proteins, adipokines and inflammatory biomarkers [14]. Although it has been known for several years that insulin can be measured in saliva, most of the studies conducted measured salivary insulin levels in the fasted state [15] and following an oral glucose tolerance test [16,17]. Results are generally supportive that saliva insulin may serve as an adequate surrogate to blood insulin levels, although the absolute concentration is lower and there may be a lag in salivary insulin changes when compared to blood in response to glucose ingestion.

To date, it is known that salivary insulin increases following the consumption of a glucose load or a mixed meal high in carbohydrates [18,19]. Pasic et al. also assessed salivary insulin levels following mixed-meals but only in individuals with type 1 diabetes who were on exogenous insulin, which makes the conclusions difficult to apply to other populations [20]. These studies support the notion that saliva insulin may reflect blood insulin levels, but do not provide insight into whether changes in saliva insulin are sensitive enough to delineate between meals with different insulin responses or whether subtle differences in postprandial insulin, which might reflect systemic insulin resistance or increased metabolic risk, are detectable via saliva insulin measures. Such information is needed to determine if saliva could be a non-invasive means to accurately measure postprandial insulin and potentially identify individuals at risk for type 2 diabetes due to hyperinsulinemia.

Accordingly, the primary aim of this study was to determine if saliva insulin could be used to delineate between postprandial insulin levels following the ingestion of low- and high-carbohydrate mixed meals designed to elicit low and high insulin responses, respectively. A secondary aim was to compare young lean participants to young overweight/obese participants to determine if subtle differences in postprandial hyperinsulinemia could be detected in saliva.

## 2. Methods

### 2.1. Participants

Sixteen individuals were recruited through poster advertisement and word of mouth across the University campus. Based on the World Health Organization (WHO) guidelines [21] eight individuals were classified as normal weight (NW) (BMI 20.0–24.9 kg/m^2^ with a waist to hip ratio <0.90 male or <0.85 female) and eight classified as overweight/obese (OO) (BMI ≥ 28.0 kg/m^2^ with a waist to hip ratio ≥0.90 male or ≥0.85 female). One female participant classified as obese was excluded from the analysis because of abnormally elevated insulin levels (values were >2.2 times the interquartile range above the median resulting in severe skewness with her data included). All participants met the following eligibility criteria: (1) being between 20 and 39 years of age; (2) not diagnosed with any medical conditions; (3) not taking any medications known to impact metabolism (on stable oral contraceptive pills for at least 3 months was accepted); (4) not following a vegetarian or low-carbohydrate high-fat diet; and (5) not a competitive athlete or participating in structured endurance training. All subjects gave their informed consent for inclusion before they participated in the study. The study was conducted in accordance with the Declaration of Helsinki, and the protocol was approved by the Ethics Committee of the UBC Clinical Research Ethics Board (H1502638) and registered at ClinicalTrials.gov (NCT02699203). Participants’ baseline characteristics are summarized in Table 1.

### 2.2. Study Design

The study followed a randomized crossover design. Randomization was performed using the online research randomizer program accessible at: https://www.randomizer.org. Eligible participants completed two isocaloric meal conditions separated by at least 72 h: (1) low-carbohydrate (LC) breakfast meal; (2) high-carbohydrate (HC) breakfast meal.

### 2.3. Study Protocol

Visit 1: After the eligibility criteria were confirmed and informed consent obtained anthropometrics and blood pressure measurements were collected. Participants were given a dietary journal to record all the food and drinks consumed for the 24-h prior to their first experimental condition. During this 24-h period, participants were instructed not to exercise and to follow their typical eating patterns such that replication would be easily accomplished on the day preceding their second experimental condition. Visit 1 occurred 2–10 days prior to Visit 2.

Visits 2 and 3: After an overnight (>10 h) fast, participants arrived at the laboratory where the research coordinator reviewed the dietary journal and confirmed that no exercise was performed in the previous 24 h. If there were no irregularities, an indwelling venous catheter was inserted by a certified phlebotomist in the antecubital space of the arm. Fasting blood and saliva samples were then collected followed by the consumption of the meal, which was consumed within 10 min. Five minutes before the second sampling time point participants were asked to rinse their mouth with water to remove any food remnants. Blood and saliva samples were then collected at 15, 30, 60, 90 and 120 min following meal completion. Following visit 2 participants were provided with their 24-h diet record and given instructions to follow their meal plan exactly prior to the next visit. On the morning of visit 3 (3–10 days following visit 2) the research coordinator reviewed the 24-h dietary journal for compliance, and confirmed that no exercise had been performed on the day before. Participants then went through the same procedures as visit 2 but consumed the alternate meal.

### 2.4. Meals

The LC meal (10% carbohydrate, 65% fat, 25% protein) was composed of whole eggs, egg whites, avocado, red peppers and onions while the HC meal (55% carbohydrate, 20% fat, 25% protein, Glycemic index: 48) [22] was composed of plain rolled oats, mixed berries (blueberries, raspberries, strawberries) and stevia sweetened whey protein isolate. Both mixed meals were isocaloric (500 kcal) and were designed to reflect food typically eaten at breakfast that would elicit a low and high insulin response respectively.

### 2.5. Blood and Saliva Sample Collection and Processing

Repeated blood samples were collected in 4 mL EDTA tubes (BD Vacutainer, Franklin Lakes, NJ, USA) using an intravenous catheter (BD Nexiva, Sandy, UT, USA). Saliva samples were collected using a passive drool collection device for a period of 60 s (Salimetrics LLC, State College, PA, USA). Both samples at each corresponding time point were kept on ice and then centrifuged together within 20 min (1550 g, 15 min, 4 °C). Plasma was immediately stored with the centrifuged saliva samples at −20 °C prior to analyses. For saliva analyses, samples were first thawed and then centrifuged again (1550 g, 15 min, 4 °C) and the clarified supernatant used for insulin analysis.

### 2.6. Biochemical Analyses

Plasma glucose was measured by the hexokinase method on a clinical chemistry analyzer (Chemwell 2910, Awareness Technologies, Ramsey, MN, USA). Plasma and salivary insulin were measured in duplicate by ELISA following the manufacturer’s protocol (Mercodia Ultrasensitive Insulin ELISA) with absorbance read on a microplate reader (iMark, Bio-Rad, Hercules, CA, USA). The coefficient of variation for duplicate samples was 10.7% for plasma insulin and 6.0% for salivary insulin. Although a previous study has used ELISA to assess fasting saliva insulin [15] we first performed validation experiments and preliminary testing revealed that there was interference in the assay with neat saliva. Spike and recovery tests showed 80 ± 7% recovery (*n* = 6) when saliva was diluted 1:2 with the zero standard provided in the kit. Diluting more than this did not appreciably increase recovery (80–85%) and tended to result in samples with low insulin concentration (e.g., lean fasting) to be below the detection limit. Therefore, 1:2 diluted saliva was used in the ELISA.

### 2.7. Statistics

Data was analyzed using SPSS v.22 (SPSS Inc., Chicago, IL, USA). Normality was assessed using Q-Q plots and Shapiro-Wilk test within each group and each meal. Appropriate transformation (natural log or 1/square root) on non-normally distributed variables resulted in normal distribution. Baseline differences were assessed using an unpaired Student *t*-test. Baseline fasting glucose, plasma insulin and salivary insulin level were computed using the average of both the LC and HC conditions for each participant. Area under the curve (AUC) and incremental AUC (iAUC) were calculated using GraphPad Prism v.6.0 (GraphPad Software Inc., San Diego, CA, USA). A two factor (group × meal) mixed ANOVA with repeated measures on the second factor was used to analyze AUC and iAUC for plasma glucose, plasma insulin, and saliva insulin. Significant interactions were followed up with pre-planned contrasts comparing NW to OO within meal and LC to HC meals within groups using Bonferroni corrections for multiple comparisons. Cohen’s d effect size was calculated for all of the pre-planned comparisons. Potential relationships between salivary and plasma AUC following both meals were assessed using separate Pearson correlations. The relationship between fasting saliva and plasma insulin levels was assessed using Spearman rank-order correlation for non-normal data. Significance was set at *p* < 0.05.

## 3. Results 

All participants complied with replication of their diet and refrained from exercising for the 24-h period preceding each experimental condition. Baseline characteristics are presented in Table 1. As expected, the OO group had a higher body mass index (BMI) and waist to hip ratio (WHR) (both *p* < 0.001). The OO group also had a significantly higher systolic blood pressure, fasting blood glucose, fasting plasma insulin and fasting salivary insulin (all *p* < 0.05). There were no differences between the two groups in terms of age, resting heart rate and diastolic blood pressure.

**Figure 1:**
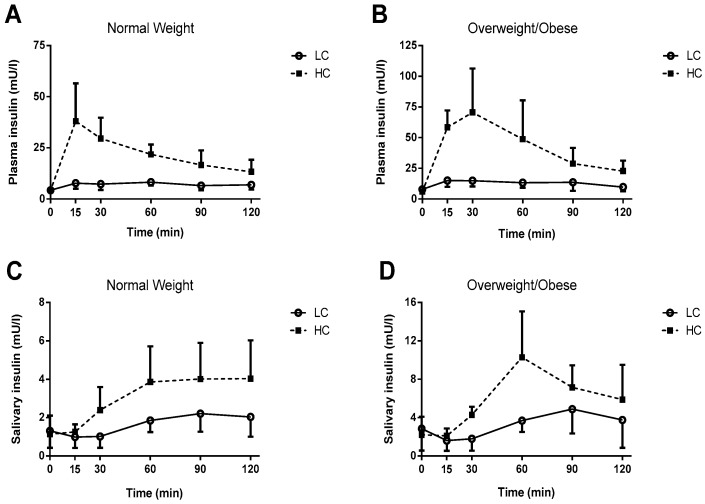
Two-hour plasma and saliva insulin responses to low-carbohydrate (LC) and high-carbohydrate (HC) breakfast meals in normal weight (NW) and overweight/obese (OO) participants. (**A**) Plasma insulin levels in NW participants; (**B**) Plasma insulin levels in OO participants; (**C**) Saliva insulin levels in NW participants; and (**D**) saliva insulin in OO participants. Statistical analyses were performed on the areas under the curve shown in Figure 2.

**Figure 2:**
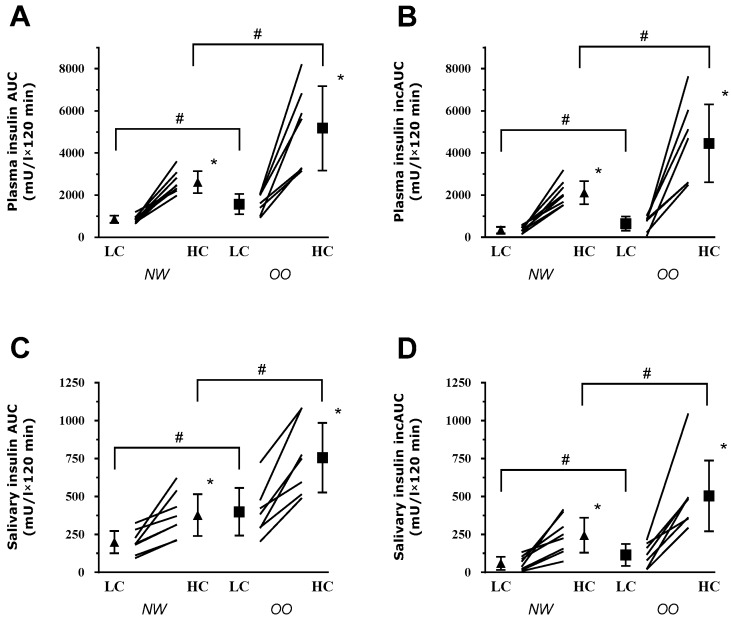
Plasma and saliva insulin area under the curve (AUC) in normal weight (NW) and overweight/obese (OO) following low-carbohydrate (LC) and high-carbohydrate (HC) breakfast meals. (**A**) Plasma AUC in NW and OO participants; (**B**) Plasma incremental AUC (iAUC) in NW and OO participants; (**C**) Saliva AUC in NW and OO participants; and (**D**) Saliva iAUC in NW and OO participants. * *p* ≤ 0.005 vs. LC meal within group. ^#^ *p* < 0.05 vs. NW group within meal.

Saliva and Plasma Insulin: The saliva and plasma insulin responses to the meals for both groups are presented in Figure 1. Total and incremental AUC are shown in Figure 2. Significant Meal × Group interactions were observed for salivary insulin AUC (*p* = 0.025), plasma insulin AUC (*p* = 0.014), salivary insulin iAUC (*p* = 0.036) plasma insulin iAUC (*p* = 0.015).

Comparison of LC to HC meals within groups: In the lean group, salivary insulin AUC (by ~89%; *p* = 0.005, d = 1.7), plasma insulin AUC (by ~205%; *p* < 0.001, d = 3.7), salivary insulin iAUC (by ~307%; *p* = 0.002, d = 2.0) and plasma insulin iAUC (by ~519%; *p* < 0.001, d = 3.4) were higher after HC as compared to LC. In the OO group, salivary insulin AUC (by ~90%; *p* = 0.001, d = 2.7), plasma insulin AUC (by ~230%, *p* = 0.002, d = 3.2) salivary insulin iAUC (by ~340%; *p* = 0.003, d = 2.5) and plasma insulin iAUC (by ~582%; *p* = 0.002, d = 2.5) were also higher after HC as compared to LC.

Comparison of OO to NW groups within meals: Salivary insulin AUC (by ~100%, *p* = 0.003, d = 1.9), plasma insulin AUC (by ~98%, *p* = 0.014, d = 2.0) salivary insulin iAUC (by ~106%, *p* = 0.022, d = 1.4) and plasma insulin iAUC (by ~111%, *p* = 0.020, d = 1.8) were significantly higher in the OO group as compared to the NW group after the HC breakfast. After the LC breakfast, salivary insulin AUC (*p* = 0.010, d = 1.6) and plasma insulin AUC (*p* = 0.007, d = 2.2) were significantly higher by ~100% and ~83%, respectively, in the OO group as compared to the NW group. Plasma insulin iAUC and salivary insulin iAUC, despite being higher in the OO group compared to the NW group after LC, did not reach statistical significance (respectively *p* = 0.067, d = 1.2 and *p* = 0.119, d = 0.9).

**Figure 3:**
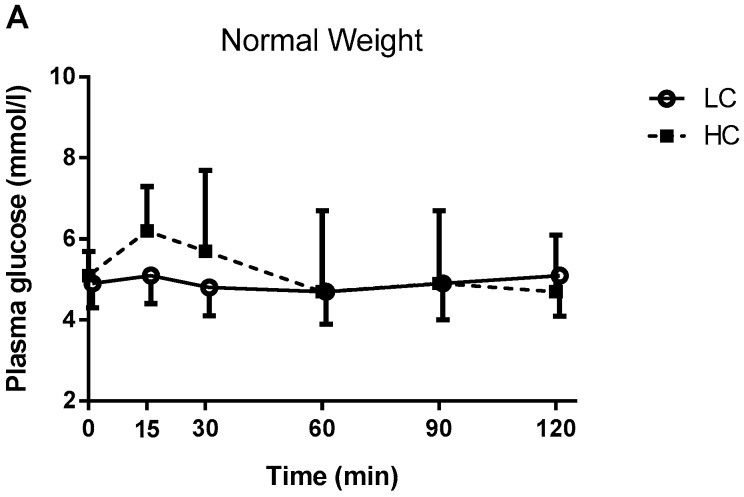

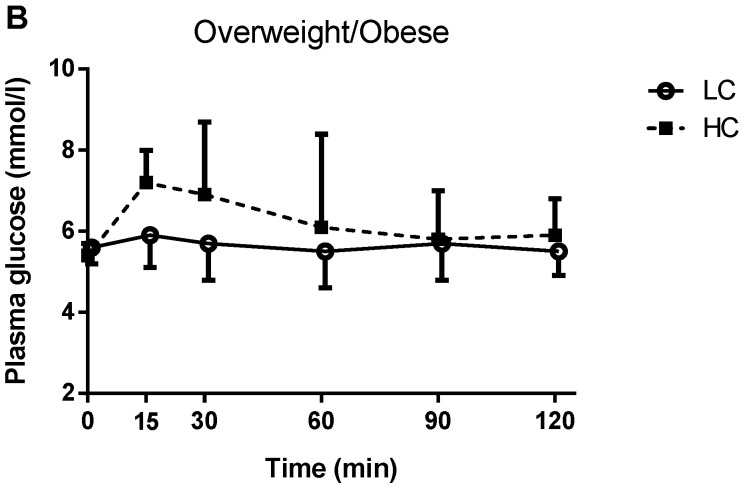
Two-hour plasma glucose responses to low-carbohydrate (LC) and high-carbohydrate (HC) breakfast meals in normal weight (NW) and overweight/obese (OO) participants. (**A**) Plasma glucose in NW participants; (**B**) Plasma glucose in OO participants.

Plasma glucose: The plasma glucose responses to the meals for both groups are shown in Figure 3. No Meal × Group interactions were found for plasma glucose AUC (*p* = 0.436) (NW; HC: 618 ± 189 vs. LC: 586 ± 94, OO; HC: 751 ± 156 vs. LC: 678 ± 94) or plasma glucose iAUC (*p* = 0.261) (NW; HC: 64 ± 108 vs. LC: 21 ± 32, OO; HC: 117 ± 118 vs. LC: 33 ± 42). The main effect of meal for plasma glucose AUC approached statistical significance (HC; 680 ± 182 vs. LC; 629 ± 102, *p* = 0.057). However, there was a significant main effect of meal for plasma glucose iAUC (HC; 89 ± 112 vs. LC; 27 ± 36, *p* = 0.012) indicating higher values after the HC meal compared to the LC meal, as expected. There were no significant differences between the NW or OO groups for plasma glucose AUC (*p* = 0.296) and iAUC (*p* = 0.122).

**Figure 4:**
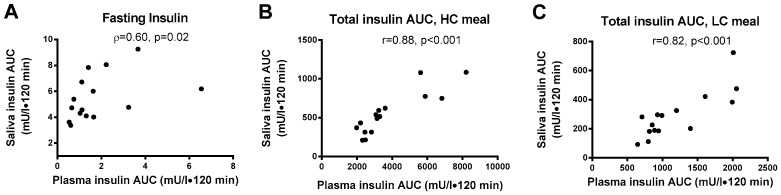
Relationships between plasma and saliva insulin concentrations. (**A**) Fasting plasma and saliva insulin; (**B**) Two-hour plasma and saliva insulin total area under the curve (AUC) following the high-carbohydrate (HC) meal; (**C**) Two-hour plasma and saliva AUC following the low-carbohyrate (LC) meal.

Relationships between plasma and saliva insulin: The fasting saliva:plasma insulin ratio was 1:3.6 in NW and 1:2.8 in OO. Fasting plasma insulin and fasting saliva insulin showed a significant positive correlation (ρ = 0.602, *p* = 0.017) (Figure 4A). Plasma insulin AUC was significantly correlated with saliva insulin AUC after the LC meal (*r* = 0.821; *p* < 0.001) and the HC meal (*r* = 0.882; *p* < 0.001) (Figure 4B,C). Saliva flow rate over 60 s was not significantly different across each time points or between meals and groups.

Peak insulin values: The peak salivary and plasma insulin values are presented in Table 2. Significant Meal × Group interactions were observed for peak salivary insulin (*p* = 0.012) and peak plasma insulin (*p* = 0.008).

Comparison of LC to HC meals within groups: In the lean group, peak salivary insulin (*p* = 0.008) as well as peak plasma insulin (*p* = 0.001) were higher after HC as compared to LC. In the OO group, peak salivary insulin (*p* = 0.001) and peak plasma insulin (*p* < 0.001) were also higher after HC as compared to LC.

Comparison of OO to NW groups within meals: Peak salivary insulin (*p* = 0.028) and peak plasma insulin (*p* < 0.001) were significantly higher in the OO group as compared to the NW group after the LC breakfast. After the HC breakfast, peak salivary insulin (*p* = 0.004) and peak plasma insulin (*p* = 0.002) were also significantly higher in the OO group as compared to the NW group.

## 4. Discussion

The objective of this study was to verify if salivary insulin could be used as a tool to delineate between high and low insulin levels following the ingestion of a high- or low-carbohydrate mixed meal. Our results consistently demonstrated that expected differences in saliva insulin responses to meals were evident; the HC meal led to larger postprandial insulin responses compared to the LC meal and OO participants had higher responses than NW. Fasting saliva insulin was also higher in OO than NW participants and significant correlations between saliva and plasma insulin were found for fasting insulin and the insulin AUC after both the HC and LC meal.

Obesity is generally accompanied by metabolic impairments including insulin resistance and hyperinsulinemia [23]. In accordance with Marchetti et al. [16], the present study showed that fasting insulin differences between groups were detectable in both plasma and saliva samples with higher levels observed in the OO as compared to NW (See Table 1). Fasting saliva insulin concentration was ~30% of the plasma concentration with absolute mU/L values slightly higher, but in the same general range, to those previously recorded in adults without diabetes [16,18,19]. Small differences in fasting saliva insulin levels could be due to the fact that these earlier studies used a radioimmunoassay technique whereas we used ultrasensitive ELISA. We also discovered interference in the insulin ELISA with undiluted saliva, a phenomenon that did not appear to be tested in these previous studies. Nonetheless, fasting saliva and plasma insulin were positively correlated (ρ = 0.60, *p* = 0.017) indicating individuals with higher basal plasma insulin levels also had higher fasting concentration of saliva insulin. A correlation of *r* = 0.92 was previously observed in the fasting state by Fabre et al., 2012 using a larger sample of overweight adolescents [15]. The stronger correlation observed in this study might be due to the difference in sample size (*n* = 277 vs. *n* = 15) and/or the population (children vs. adults). The differences between OO and NW groups in fasting saliva (and plasma) insulin were seen despite both groups having glucose levels in the normoglycemic range, highlighting the potential utility of saliva insulin for detecting underlying insulin resistance.

Significant Meal × Group interactions were observed for 2-h plasma and salivary insulin AUC. Since differences in baseline (fasting) insulin levels were detected, we also computed the respective iAUC, which also revealed similar statistically significant meal × group interactions. Cohen’s d effect sizes for pairwise comparisons were large to very-large (0.9 to 3.7) and confirmed the magnitude of the differences observed between low-carbohydrate and high-carbohydrate meals as well as the NW and OO groups. Carbohydrates are known as the most potent stimulator of insulin secretion [24] with increased secretion observed when combined to insulinotropic amino acids [25]. In our study, the HC meal increased the total and incremental insulin AUC over the 2-h postprandial period as compared to LC in both groups with a greater increase in the OO as compared to NW. Importantly, these differences were similarly observed in both plasma and saliva samples suggesting that expected differences in insulin levels following the consumption of distinct meals can potentially be tracked in the saliva. Previously Fekete et al. and Messenger et al. showed that salivary insulin was increased following a mixed meal tolerance test in lean and overweight individuals (body mass index 21–28 kg/m^2^) [18,19]. However, the effects of body mass index on salivary and plasma insulin levels were not assessed in these studies nor were different meals compared. Thus, our observations add to the limited literature on saliva insulin monitoring by showing that differences in salivary insulin can be tracked between meals with different insulinogenic effects and between NW and OO participants.

It is of interest to note the significant correlations between plasma and salivary insulin total AUC (LC: *r* = 0.821 and HC *r* = 0.882). Positive linear correlations varying from 0.50 to 0.91 have previously been observed after both an oral glucose tolerance or single meal tolerance tests in healthy adults, obese individuals and people with both type 1 and type 2 diabetes [16,20,26]. Generally speaking, the peak insulin response in saliva was delayed by 30–45 min relative to blood, although without traceable insulin and more frequent sampling we were not able to quantify the exact time lag for peak insulin from blood to saliva. In line with our findings, a 15 to 60 min delay in the peak concentration of salivary insulin has consistently been reported by other groups following a mixed meal [18,19] and an oral glucose load [16,20].

No interactions or group effect were observed in terms of glucose AUC and iAUC. However, we observed a MEAL effect for the postprandial glucose iAUC (*p* = 0.012) while the AUC approached significance level (*p* = 0.057). The absence of glucose differences between groups combined with the higher insulin levels in OO as compared NW suggests that the OO group was able to successfully compensate for a higher degree of insulin resistance by increasing insulin secretion. Moreover, these results suggest that some metabolic impairments and underlying insulin resistance are present, and can be detected with both plasma and saliva insulin measurements, in overweight to obese but otherwise healthy, young and normoglycemic participants.

This study has some limitations that should be acknowledged. First, the relatively small sample size limit our ability to fully appreciate the range of achievable postprandial saliva insulin levels in healthy adults. Second, our results are limited to the insulin and glucose responses in the fasting state and following a morning mixed meal, which might differ from meals consumed at other times of the day. Finally, more frequent samples along with a longer postprandial period (e.g., 3–4 h) would have been useful in order to better compute the insulin curves and determine the timing of when saliva insulin returns to basal levels. Despite the above limitations, our study is strengthened by the fact that we used a randomized crossover design for the meal intervention and assessed metabolic responses to “real-life” mixed-meals.

In conclusion, this study provides evidence that saliva could potentially be used to delineate between high and low insulin levels in both the fasting state and following mixed meals. Additional studies using larger sample sizes are warranted to better explore the kinetics and consistency of salivary insulin responses following mixed meals, to further validate this non-invasive assessment of metabolic health.

## Figures and Tables

**Table 1:** Baseline participants’ characteristics.

Outcomes	Normal Weight	Overweight/Obese	*p* Value
Number of participants (M/F)	8 (5/3)	7 (6/1)	-
Age (years)	27.1 (4.1)	30.6 (4.3)	0.133
Body mass index (kg/m^2^)	22.4 (1.8)	31.0 (1.8)	<0.001
Waist to Hip ratio (cm)	0.79 (0.06)	0.93 (0.06)	<0.001
Systolic blood pressure (mmHg)	118 (7)	129 (12)	0.041
Diastolic blood pressure (mmHg)	76 (8)	83 (7)	0.072
Resting heart rate (bpm)	62 (13)	63 (10)	0.929
Fasting glucose (mmol/L)	5.0 (0.5)	5.5 (0.3)	0.046
Fasting plasma insulin (mU/L)	4.3 (0.7)	7.0 (1.6)	0.003
Fasting saliva insulin (mU/L)	1.2 (0.9)	2.5 (2.0)	0.039

**Table 2:** Insulin peak values following the ingestion of mixed meal breakfasts in normal weight and overweight/obese participants.

Outcomes	Normal Weight		Overweight/Obese	
	Range	Mean (SD)	Range	Mean (SD)
Low-carbohydrate meal				
Peak saliva insulin (mU/L)	1.0–3.9	2.5 (1.1)	2.9–9.9	5.7 (2.9) *
Peak plasma insulin (mU/L)	7.6–13.3	9.7 (2.1)	11.8–23.9	18.7 (4.1) *
High-carbohydrate meal				
Peak saliva insulin (mU/L)	2.5–8.2	4.8 (2.2) ^#^	6.4–16.8	10.8 (4.3) *^,#^
Peak plasma insulin (mU/L)	22.1–61.8	39.9 (15.8) ^#^	47.5–125.8	81.8 (26.1) *^,#^

Meal × Group interactions were significant for all insulin outcome (*p* < 0.05). * Significantly different from normal weight within meal. ^#^ Significantly different from low-carbohydrate meal within group.
